# Clinical–Epidemiological Profile of COVID-19 Patients Admitted during Three Waves of the Pandemic in a Tertiary Care Center, in Belém, Pará, Amazon Region of Brazil

**DOI:** 10.3390/v16081233

**Published:** 2024-07-31

**Authors:** Ana Beatriz Nunes Pereira, Fernando Sérgio Henriques Pereira, Júlio Éden Davi Lopes Araújo, Rangel Pereira Brasil, Angélica Menezes Bessa Oliveira, Sandra Souza Lima, Ricardo Roberto de Souza Fonseca, Rogério Valois Laurentino, Aldemir Branco Oliveira-Filho, Luiz Fernando Almeida Machado

**Affiliations:** 1 Biology of Infectious and Parasitic Agents Post-Graduate Program, Federal University of Pará, Belém 66075-110, PA, Brazil; anabianunes.fisio@gmail.com; 2 Virology Laboratory, Institute of Biological Sciences, Federal University of Pará, Belém 66075-110, PA, Brazil; saraujo@ufpa.br (S.S.L.); ricardofonseca285@gmail.com (R.R.d.S.F.); valois@ufpa.br (R.V.L.); 3 Belém Health Department, Humberto Maradei Pereira Municipal and Emergency Hospital, Belém 66075-259, PA, Brazil; dinhopsiq@gmail.com (F.S.H.P.); julioeden@gmail.com (J.É.D.L.A.); rangelbrasilp@yahoo.com.br (R.P.B.); angelbssa@hotmail.com (A.M.B.O.); 4 Study and Research Group on Vulnerable Populations, Institute for Coastal Studies, Federal University of Pará, Bragança 68600-000, PA, Brazil; olivfilho@ufpa.br

**Keywords:** COVID-19, SARS-CoV-2, new coronavirus including SARS-CoV-2, epidemiology of opportunistic viral infections, patient outcome assessments

## Abstract

Background: Coronavirus disease 2019 (COVID-19) is a disease with a broad clinical spectrum, which may result in hospitalization in healthcare units, intensive care, and progression to death. This study aimed to describe and compare the clinical and epidemiological profile of COVID-19 during the three waves of the disease, in patients admitted to a public hospital in the city of Belém, Pará, in the Amazon region of Brazil. Methods: This descriptive, observational, and cross-sectional study was population-based on individuals who were hospitalized with a diagnosis of COVID-19, confirmed by real-time reverse transcription-polymerase chain reaction (RT-PCR), and who were interviewed and monitored at the public hospital, from February 2020 to April 2022. Results: The prevalence was male patients, older than 60 years. The most frequent symptoms were dyspnea, cough, and fever. Systemic arterial hypertension was the most prevalent comorbidity followed by diabetes mellitus. Less than 15% of patients were vaccinated. The nasal oxygen cannula was the most used oxygen therapy interface followed by the non-rebreathing reservoir mask. Invasive mechanical ventilation predominated and the median time of invasive mechanical ventilation ranged from 2 to 6 days among waves. As for the hospital outcome, transfers prevailed, followed by deaths and discharges. Conclusion: The presence of comorbidities, advanced age, and male sex were important factors in the severity and need for hospitalization of these patients, and the implementation of the vaccination policy was an essential factor in reducing the number of hospital admissions.

## 1. Introduction

Coronavirus disease 2019 (COVID-19) is a disease with a broad clinical spectrum. Infection with SARS-CoV-2 can result in a range of presentations, from asymptomatic to critical illness requiring hospitalization and treatment in an intensive care unit (ICU), and even death [1]. The incubation period of the disease typically ranges from 5 to 13 days. However, it is not well defined in asymptomatic cases [2,3]. Most COVID-19 infections present with mild or moderate symptoms. This milder form typically has a good prognosis, does not require hospitalization, and is more common in young adults without underlying health conditions [4,5].

Severe cases progress to more severe respiratory symptoms, including dyspnea, hypoxemia requiring oxygen therapy, and significant lung injuries. Critical cases experience a further escalation, with severe hypoxemia, respiratory failure necessitating invasive mechanical ventilation (IMV), coagulopathies, encephalopathies, acute kidney injury, multiple organ failure, shock, and ultimately death [6,7]. In general, both severe and critical cases present extensive and important radiological changes, along with laboratory abnormalities such as low albumin levels, hyperbilirubinemia, leukopenia or leukocytosis, lymphopenia, eosinopenia, and elevated levels of C-reactive protein (CRP), lactic dehydrogenase, creatinine, and liver enzymes [8]. Worsening of the condition is associated with the elderly and comorbidities such as immunosuppression, heart disease, lung disease, and diabetes, and there is no good prognosis [9].

Globally, over 770 million SARS-CoV-2 infections have been confirmed, tragically resulting in more than 6.9 million deaths. The infection incidence rate stands at 82.79 per 100,000 inhabitants, while the mortality rate is 8.39 per 100,000 inhabitants [10]. Brazil ranks sixth with the highest number of cases (37.72 million), following the United States (103.44 million), China (99.32 million), India (45.00 million), France (38.99 million), and Germany (38.44 million) [11]. Regarding cases of severe acute respiratory syndrome, since the beginning of the pandemic, 3.516.381 cases have been recorded, totaling 860.483 deaths, with 2.169.470 of the cases resulting from COVID-19, resulting in 682.725 deaths [12].

The northern region of the country has been severely impacted, with over 2.9 million confirmed cases, more than 51,000 deaths, an incidence rate exceeding 15,000 per 100,000 inhabitants, and a mortality rate surpassing 280 per 100,000 inhabitants [13]. Pará, within the northern region, has also seen a significant burden, exceeding 880,000 confirmed cases and over 19,000 deaths. The incidence rate in Pará is higher than 10,200 per 100,000 inhabitants, and the mortality rate surpasses 220 per 100,000 inhabitants [13].

According to the Coronavirus in Pará portal, provided by Pará’s State Health Secretary, regarding the epidemiological profile of the COVID-19 infection in Pará State, cases were most prevalent among females (55.7%), although the death rate was higher in males (58.7%). The 30–39 age group had the highest number of cases, while the 70–79 age group had the highest death rate [14]. The main symptoms reported were cough and fever, followed by sore throat, headache, runny nose, shortness of breath (dyspnea), muscle or joint pain (myalgia/arthralgia), nausea/vomiting, weakness (adynamia), nasal congestion, chills, and others. The most important comorbidities were heart disease and diabetes [14].

Socioeconomic and demographic heterogeneity in Brazil is directly reflected in the distribution and quality of public health services and availability of hospital and ICU beds; mainly, in the Brazilian northern region where services towards specific populations such as pregnant and puerperal women are scarce, this leads to a great negative impact on health indicators during pandemics such as COVID-19, which may cause a decrease in pregnant and puerperal women’s prognoses. Despite the abundance of epidemiological data in Brazil, few articles explore the comparison between waves, comparing demographic and clinical characteristics and disease progression among pregnant women and puerperal women hospitalized with COVID-19. Therefore, the present study aimed to describe and compare the clinical and epidemiological profile of COVID-19 during the three waves of the disease, in patients admitted to a public hospital in the city of Belém, Pará, in the Amazon region of Brazil from 2020 to 2022.

## 2. Materials and Methods

### 2.1. Type of Study and Ethical Aspects

This descriptive, observational, and cross-sectional study was population-based on individuals who were hospitalized with a diagnosis of COVID-19, confirmed by real-time reverse transcription-polymerase chain reaction (RT-PCR) (Thermo Fisher, Waltham, MA, USA), and who were interviewed and monitored at the Hospital e Pronto Socorro Municipal Humberto Maradei Pereira (HPSM-HMP), from 18 February 2020 to 20 April 2022. The study was conducted and approved by the Research Ethics Committee of the Municipal Health Department (SESMA), under protocol number 5.822.850, and all participants were included in the study after providing written consent.

### 2.2. Study Population and Data Collection

The study eligibility criteria were (i) individuals with a confirmed diagnosis of SARS-CoV-2 infection; (ii) having all clinical signs and symptoms associated with SARS-CoV-2 infection; (iii) admitted at the HPSM in Belém, Pará; (iv) medical records duly filled in; (v) have any kind of comorbidity; (vi) age ≥ 1 year old; (vii) resident of Pará state or resident during COVID-19 epidemiological waves; (ix) signed informed consent terms. The exclusion criteria were (i) individuals who had not agreed to sign the consent terms; (ii) medical records not filled out correctly; (iii) duplicity of patients seeking care in HPSM during different COVID-19 epidemiological waves; (iv) have any type of impairment that excluded patients from this study. 

After the inclusion criteria, the COVID-19 symptoms’ evaluation, SARS-CoV-2 infection confirmation, and mortality risk for each participant was allocated in three groups: G1: hospitalized patients treated admitted in the first wave; G2: hospitalized patients admitted in the second wave; and G3: hospitalized patients admitted in the third wave. Epidemiological, sociodemographic, and clinical data were collected by a single trained #2 healthcare provider previously calibrated by the Kappa test and with previous experience in clinical studies.

The data were collected from information available in the medical records and clinical examination: age range, municipality, all types of comorbidities, COVID-19 vaccination status, admission symptoms such as cough, sore throat or runny nose, anosmia, ageusia, diarrhea, abdominal pain, fever, chills, myalgia, fatigue, headache, adynamia, prostration, hyporexia, diarrhea, pneumonia, SARS, dyspnea, persistent chest pressure, O2 saturation below 95%, bluish coloration of the lips or face, the need for invasive mechanical ventilation or oxygen therapy and its duration, and patients’ outcomes such as discharge, transfer (if the patient was transferred to other units, the follow-up of this study still went on in order to maintain study viability and credibility), and death.

The COVID-19 waves were divided according to its peaks in Pará State, according to data from Pará’s State Health Secretary; therefore, this study comprised three waves defined as follows: Wave 1: 18 February 2020–10 September 2020 (peak: 5 May 2020); Wave 2: 20 October 2020–2 September 2021 (peak: 15 March 2021); Wave 3: 31 October 2021–15 April 2022 (peak: 22 January 2022) [11].

### 2.3. Statistical Analysis

The data were stored and tabulated in Microsoft Excel^®^ 2019 (Microsoft Corporation) spreadsheets and the graphs were produced in GraphPad Prism 10 (GraphPad Software Inc., San Diego, CA, USA). Statistical analysis was performed using Bioestat 5.3^®^ (Sociedade Civil Mamirauá: Belém, Pará, Brasil). All numerical variables were assessed for normality using the Kolmogorov–Lilliefors test. To compare categorical variables, the chi-square test with residual analysis and the G test were used, according to the characteristics of the variables. For the independent numerical variables, the Mann–Whitney and Kruskal–Wallis tests and Dunn post-test were applied, as they did not admit normality. For paired numerical variables, the Wilcoxon test was used. The significance level adopted was 5% (*p* < 0.05).

## 3. Results

Initially, 740 patients were screened during the study period. Of these, 417 were excluded (i) because they were not admitted to the hospital (*n* = 347), (ii) because they left the hospital (*n* = 20), or (iii) because they did not agree to participate in the research (*n* = 50). Thus, 323 patients without age and sex restrictions were included and admitted to the hospital with a confirmed diagnosis of COVID-19.

This study examined the age and sex distribution of participants across three groups (G1, G2, and G3). G1 had the highest prevalence of individuals aged 60–69 years (27.3%), followed by 40–49 years old (21.7%). In contrast, G2 primarily consisted of participants aged 70–79 years (32.3%), with a secondary peak at 60–69 years (20%). Interestingly, G3 demonstrated a more even distribution between these two age groups (60–69 years: 23.8% and 70–79 years: 22.6%). Statistical analysis revealed significant differences in age distribution between G1 and both G2 (*p* = 0.0042) and G3 (*p* = 0.0021). Regarding sex, males dominated G1 (56.6%) and G2 (54.6%), whereas G3 had a slight female majority (54.0%). However, there was no statistically significant difference in sex distribution across all groups (Table 1).

The most frequently reported symptoms in G1 were dyspnea (82.5%), cough (67.8%), and fever (64.3%). In G2, the same symptoms prevailed, but in a lower percentage, being 65.4%, 59.2%, and 50.7%, respectively. In G3, there was also a prevalence of dyspnea (52%), fever (50%), and cough (44%), while there were no reports of arthralgia, anosmia, or ageusia. The symptoms that showed a significant difference between G1 and G2 were dyspnea (*p* = 0.0019), fever (*p* = 0.0320), diarrhea (*p* = 0.0033), myalgia (*p* = 0.0308), ageusia (*p* = 0.0167), and cardiological symptoms (*p* = 0.0215). Between G1 and G3, the significant symptoms were dyspnea (*p* < 0.0001), cough (*p* = 0.0049), desaturation (*p* = 0.0005), myalgia (*p* = 0.0010), anosmia (*p* = 0.0185), musculoskeletal symptoms (*p* = 0.0232), neurological symptoms (*p* = 0.0050), and cardiological symptoms (*p* = 0.007). And between G2 and G3, the differences were significant for desaturation (*p* = 0.0010), anosmia (*p* = 0.0095), and ageusia (*p* = 0.0140).

Systemic arterial hypertension (SAH) was the most prevalent comorbidity in the three groups (52.4%, 47.7%, and 58%, respectively), followed by diabetes mellitus (DM) with 30.8%, 38.5%, and 34%, respectively. In G3, there were no reports of asthma, Alzheimer’s, or lung diseases. The significant intergroup differences, between G1 and G2, were Alzheimer’s (*p* = 0.0315), drug allergy (*p* = 0.0236), lung diseases (*p* = 0.0005), and neuropathies (*p* = 0.0067). Between G1 and G3, there were significant differences in Alzheimer’s (*p* = 0.0384), stroke sequelae (*p* = 0.0139), and drug allergy (*p* = 0.0009). And between G2 and G3, there was only a significant difference in the report of lung diseases (*p* = 0.0043) (Table 2).

Regarding vaccination status, G1 had 0% vaccination due to the unavailability of the immunizing agent; in G2, 15.4% were vaccinated, 11.5% with only the first dose, 3.8% with the second dose, and 84.6% had not yet taken any dose. In G3, 56% of patients had already been vaccinated, 6% with only the first dose, 36% with the second dose, 10% with the first booster dose, 4% with the second booster dose, and 44% had not yet taken the immunizer. There was a significant difference between G1 and G2 (*p* < 0.0001), G2 and G3 (*p* < 0.0001), and G2 and G3 (*p* < 0.0001) (Table 3).

The nasal oxygen cannula (CNO2) was the most used oxygen therapy interface (50.8%), followed by the non-rebreathing reservoir mask (MR) with 19.2%. In G1, CNO2 was used in 69.9% of cases; in G2, 35.4% used CNO2 and 34.6% used MR; and in G3, 36% needed CNO2 and 26% MR. In G3, no patient used continuous macro nebulization (MNC) and in Groups 2 and 3 the venturi system was not used. Between groups, there was a significant difference in CNO2 (*p* < 0.0001) and MR (*p* < 0.0001) between G1 and G2. Between G1 and G3, the difference was in CNO2 (*p* = 0.0002) and MR (*p* < 0.0001). And there was no significant difference between G2 and G3 (Table 4).

Regarding ventilatory support, invasive mechanical ventilation (IMV) predominated in the three groups (28.7%, 44.6%, and 36%, respectively), while non-invasive mechanical ventilation was not used in G1, comprising 11.5% in G2 and 2% in G3. Between G1 and G2, there was a significant difference in NIV (*p* < 0.0001), and between G1 and G3 and between G2 and G3, there was no significant difference in ventilatory support (Table 4).

**Figure 1:**
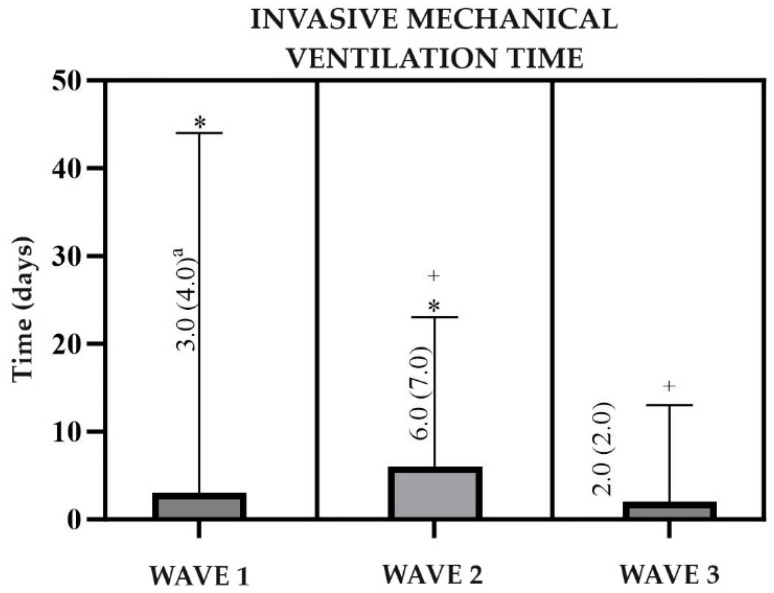
Clinical information on the time of invasive mechanical ventilation of patients hospitalized with COVID-19 at the public hospital in Belém, Pará, Brazil, from February 2020 to April 2022. ^a^ Median and interquartile range; * Significant difference in invasive mechanical ventilation time between Waves 1 and 2 (*p* < 0.0500); ^+^ Significant difference in invasive mechanical ventilation time between Waves 2 and 3 (*p* < 0.0500).

The median time on IMV was 3 (4.0) days in G1, 6 (7.0) days in G2, and 2 (2.0) days in G3. There was a significant difference between G1 and G2 (*p* < 0.0500) and between G2 and G3 (*p* < 0.0500), while between G1 and G3 there was no significant difference (Figure 1). As for the hospital outcome, in G1, transfers prevailed (58%), followed by deaths (24.5%) and discharges (17.5%). In G2, 66.9% were transferred, 20.8% died, and 12.3% were discharged. And in G3, there were 56% of transfers, 30% of deaths, and 15% of discharges. Significant differences occurred between G1 and G2 (*p* < 0.0001) and G2 and G3 (*p* = 0.0002), with no significant difference between G1 and G3 (Table 5).

## 4. Discussion

This study observed a higher prevalence of males, potentially reflecting established trends of lower self-care adherence among men. This could contribute to increased susceptibility to infections and comorbidities, ultimately leading to higher mortality rates in this demographic. Interestingly, G1 displayed a significant presence of individuals under 60 years (21.7%) alongside the dominant elderly population (60–69 years: 27.3%). This pattern may be attributable to the inclusion of younger age groups in the population’s vaccination policy, whereas the higher representation of elderly individuals likely reflects the age-related decline in physiological resilience.

These data are in line with the literature, since although COVID-19 affects individuals of all age groups and both sexes, there is a prevalence among men and those over 40 years of age. The study carried out in Northern Ireland showed a prevalence of females (59.8%) and an age group over 50 years old (median 57.1). This profile of the first infected people changed and gave rise to a prevalence of males (59%) and those aged under 50 years (median 39 years). A study carried out in Brazil demonstrated that men are more likely to suffer from COVID-19 (53.3%) than women, just as men and the elderly are at a greater risk of dying. Another study carried out in intensive care units in China found a prevalence of 63% of male patients and an average age of 64 years. The study by Sun et al. demonstrated the prevalence among men and elderly people in Waves 1 and 2 of the disease in China [15,16,17,18,19].

As for symptoms, it can be observed that the most frequent in the three groups were fever, cough, dyspnea, and other respiratory complaints, despite the gradual reduction with the passage of the waves. An increase in neurological, cardiac, and musculoskeletal complaints was also evident. This can be justified by the fact that the study population consisted of potentially serious patients who required hospitalization; therefore, both pulmonary and extrapulmonary symptoms were more frequent.

Similar data are found in the literature, since COVID-19 presents a wide variety of symptoms that are confused with other flu syndromes so the most frequently reported are the triad of fever, cough, and dyspnea. Although respiratory symptoms prevail, the disease is systemic and, consequently, causes a series of extrapulmonary symptoms that, although less frequent, deserve attention, as some are related to a more serious clinical condition and with a negative evolution, as is the case of some cardiovascular and neurological symptoms; however, the increase in non-respiratory symptoms in our study might be due to heightened awareness and reporting rather than an actual increase in incidence, so this bias was taken into consideration during our analysis [20,21].

Furthermore, around a third of patients hospitalized with COVID-19 present some type of neurological complaint, such as headache, stroke, anosmia, and an altered level of consciousness. Patients with comorbidities, mainly hypertension and age over 60 years, are more predisposed to serious infections, with fewer respiratory complaints, more neurological complaints, such as cerebrovascular symptoms and an altered level of consciousness, and musculoskeletal disorders. Cardiac symptoms are associated with increased mortality and a poor outcome. It is believed that 20 to 30% of patients hospitalized with COVID-19 have some type of cardiac dysfunction, with the common complaints being palpitation (20%) and chest pain (17%), followed by other signs and symptoms such as sinus tachycardia, bradycardia, arterial and venous thromboembolic events, and myocarditis, among others [22,23,24,25].

We can observe that hypertension and DM were the most reported comorbidities in the three groups. The mechanism by which these comorbidities are more associated with SARS-CoV-2 infection is not known for certain; therefore, treatment is a possible association, as it is carried out with medications that inhibit the angiotensin-converting enzyme 1 (ACE inhibitors) and the angiotensin receptor blockers (ARB), which can increase the expression of ACE 2 and facilitate the entry of the virus into the body. Despite this possible correlation, the interruption of treatment with ACE inhibitors and ARBs is associated with greater risks of disease severity and death. Furthermore, hypertension may be the most common, as it is the most prevalent comorbidity in the world, affecting around 30% of the world’s population [26,27,28].

Drug allergy was a comorbidity that increased significantly from G1 to G3. The literature is scarce regarding the correlation between COVID-19 and drug allergy as a comorbidity, which is why studies are more focused on adverse allergic reactions to treatment and vaccination. But when it comes to allergies in general, no correlation has been observed between the severity of symptoms and the presence of allergic diseases [29,30]. The fact that approximately 50% of patients in the three groups had comorbidities reaffirms that the presence of comorbidities is strongly related to the severity of the disease and the need for hospitalization. Research shows that 20 to 50% of individuals with COVID-19 have at least one comorbidity, with cardiovascular, chronic obstructive pulmonary, chronic renal, cerebrovascular, and oncological diseases being the most related to mortality [31].

Regarding vaccination, in G1 100% of patients were not vaccinated, but this is expected, given that the first doses of the vaccine against COVID-19 began to be administered in Brazil on 17 January 2021 [32]. In G2, the presence of vaccinated patients is already observed (15.4%); however, the percentage is still very low, and in G3, all patients should take at least the first dose of the vaccine, only 56% were vaccinated, of which 4% with the four recommended doses. Thus, vaccine hesitancy contributed to the need for hospital admission and the greater severity of the disease.

Vaccine hesitancy among pregnant women concerning COVID-19 vaccines stems from several factors, primarily driven by a lack of data at the outset. Studies like that of Alkhalifah et al. [33] detailed that misinformation about the vaccination efficacy or side effects contributed to general hesitancy, especially among pregnant women. Gupta et al. [34] highlighted concerns about the vaccines’ safety due mainly to vaccinees’ fear of a harmful effect or potential unknown effects on the fetus which fueled anxieties about it. Additionally, Dahlen et al. [35] also suggested that hesitancy can be further influenced by sociodemographic factors like age and income. We must address these concerns through open communication with healthcare providers, emphasizing the growing body of evidence on vaccine safety and efficacy in pregnancy.

This process of vaccine hesitancy has also been experienced in other parts of the world, so that at the end of October 2023, 35.1% of the world’s population had not yet taken any dose of the COVID-19 vaccine. Studies carried out in Europe and the Middle East observed that young people, women, and people of low socioeconomic status and without comorbidities were more prone to hesitation, the main justifications being the low perception of the risks that COVID-19 brings to health, low confidence in its effectiveness and safety as an immunizer against the SARS-CoV-2 virus, as well as the fear of adverse reactions, ideological reasons, and the dissemination of fake news in the media [36,37]. In Brazil, political–ideological disputes and the circulation of fake news in the media and social networks greatly influenced vaccine hesitancy and non-compliance with safety recommendations from health agencies [38,39,40].

As the main symptoms of the disease were respiratory, the use of oxygen therapy was a common practice, with the most used interface being CNO2 (50.8%), followed by the mask with a non-rebreathing reservoir (19.2%). It is worth mentioning that in G1 there was a low prevalence of MRI use, which is justified by the collapse of the health systems that occurred during the period, so that in the hospital in question there was no great availability of this interface for use. Regarding ventilatory support, IMV predominated due to the severity of the hospitalized patients. Furthermore, the median time on mechanical ventilation was shorter than that found in the literature, which is justified by the high percentage of transferred patients, which made monitoring cases difficult. These data are similar to those found in other studies in which around 80% of hospitalized individuals use at least one type of oxygen therapy, with CNO2 and MR being the most used, and around 50% require positive pressure ventilation support. The average time of IMV is conflicting in the literature, ranging from 6 to more than 30 days [41,42].

The predominant hospital outcome was transfer with 61.3% of the total, the lowest percentages of discharges and deaths occurred in G2, coinciding with the highest percentage of transfers. The highest percentage of discharges occurred in G1 and the highest percentage of deaths in G3. These data differ from the literature in that they present high hospital transfer rates, causing interruptions in monitoring the condition and consequently reducing discharge and death rates. This high transfer rate is due to the profile of the emergency hospital, which stabilizes patients and transfers them to specialized treatment units. A study carried out in a hospital that treats infectious diseases found that half of the patients were discharged, 26% died, and 25% were transferred. In a public hospital in Fortaleza, 52.8% were discharged and 47.2% died, while a hospital in the south of the country had 90% discharged and only 10% died [43]. The high transfer rates are because, during the COVID-19 pandemic, public healthcare systems were stretched at its peak, impacting essential services like prenatal care. Limited vital prenatal care resources, including staff and medical supplies, were diverted to manage the surge of COVID-19 cases. This resulted in a domino effect: pregnant women, fearing infection or facing facility closures, avoided routine checkups, causing disruptions in maternal health service use.

## 5. Conclusions

Thus, it is concluded that the presence of comorbidities, advanced age, and male gender were important factors in the severity and need for hospitalization of these patients and that the implementation of the vaccination policy was an essential factor in reducing the number of hospital admissions.

## Figures and Tables

**Table 1:** Epidemiological and symptoms information from data on patients hospitalized with COVID-19 at the public hospital in Belém, Pará, Brazil, from February 2020 to April 2022.

Parameters	G1 *n* (%)	G2 *n* (%)	G3 *n* (%)	Total *n* (%)	*p*-Value
Sex					
Male	81.0 (56.6)	71.0 (54.6)	23 (46.0)	175 (54.2)	0.1071
Female	62 (43.4)	59.0 (45.4)	27 (54.0)	148 (45.8)	
Age group					
1–9	0.0 (0.0)	0.0 (0.0)	1.0 (2.0)	1.0 (0.3)	0.0004 ^a^
10–19	1.0 (0.7)	1.0 (0.8)	1.0 (2.0)	3.0 (0.9)	
20–29	1.0 (0.7)	6.0 (4.6)	4.0 (8.0)	11.0 (3.4)	
30–39	13.0 (9.1)	8.0 (6.1)	2.0 (4.0)	23.0 (7.1)	
40–49	31.0 (21.7)	15.0 (11.5)	3.0 (6.0)	49.0 (15.1)	
50–59	16.0 (11.2)	18.0 (13.8)	3.0 (6.0)	37.0 (11.5)	
60–69	39.0 (27.3)	26.0 (20.0)	12.0 (24.0)	77.0 (23.8)	
70–79	19.0 (13.3)	42.0 (32.3)	12.0 (24.0)	73.0 (22.6)	
80–89	20.0 (14.0)	12.0 (9.2)	7.0 (14.0)	39.0 (12.1)	
90–99	3.0 (2.1)	2.0 (1.5)	5.0 (10.0)	10.0 (3.1)	
Admission symptoms					
Dyspnea	118.0 (82.5)	85.0 (65.4)	26.0 (52.0)	229.0 (70.9)	< 0.0001 *
Cough	97.0 (67.8)	77.0 (59.2)	22.0 (44.0)	196.0 (60.7)	0.004 **
Fever	92.0 (64.3)	66.0 (50.7)	25.0 (50.0)	183.0 (56.7)	0.0457 ***
Desaturation	54.0 (37.8)	47.0 (36.1)	5.0 (10.0)	106.0 (32.8)	0.0009 ****
Chest pain	31.0 (21.7)	28.0 (21.5)	6.0 (12.0)	65.0 (20.1)	0.2968
ARF ^aa^	12.0 (8.4)	7.0 (5.4)	2.0 (4.0)	21.0 (6.5)	0.4530
Odynophagia	6.0 (4.2)	9.0 (6.9)	1.0 (2.0)	16.0 (4.9)	0.3124
Inappetence	8.0 (5.6)	8.0 (6.1)	5.0 (10.0)	21.0 (6.5)	0.5763
Nausea	18.0 (12.6)	15.0 (11.5)	11.0 (22.0)	44.0 (13.6)	0.1659
Vomit	17.0 (11.9)	14.0 (10.8)	11.0 (22.0)	42.0 (13.0)	0.1160
Diarrhea	25.0 (17.5)	8.0 (6.1)	5.0 (10.0)	38.0 (11.8)	0.0121 ^#^
Abdominal pain	8.0 (5.6)	10.0 (7.7)	8.0 (16.0)	26.0 (8.0)	0.0948
Malaise	7.0 (4.9)	11.0 (8.5)	3.0 (6.0)	21.0 (6.5)	0.4877
Asthenia	15.0 (10.5)	16.0 (12.3)	8.0 (16.0)	39.0 (12.1)	0.5854
Myalgia	26.0 (18.2)	12.0 (9.2)	1.0 (2.0)	39.0 (12.1)	0.0019 ^##^
Arthralgia	3.0 (2.1)	6.0 (4.6)	0.0 (0.0)	9.0 (2.8)	0.1084
Back pain	3.0 (2.1)	8.0 (6.1)	1.0 (2.0)	12.0 (3.7)	0.1702
Edema	8.0 (5.6)	11.0 (8.5)	2.0 (4.0)	21.0 (6.5)	0.4609
Walking deficit	8.0 (5.6)	7.0 (5.4)	8.0 (16.0)	23.0 (7.1)	0.0579
Anosmia	9.0 (6.3)	10.0 (7.7)	0.0 (0.0)	19.0 (5.9)	0.0332 ^###^
Ageusia	2.0 (1.4)	9.0 (6.9)	0.0 (0.0)	11.0 (3.4)	0.0087 ^+^
Headache	19.0 (13.3)	18.0 (13.8)	3.0 (6.0)	40.0 (12.4)	0.3260
RLC ^b^	10.0 (7.0)	12.0 (9.2)	9.0 (18.0)	31.0 (9.6)	0.1021
Disorientation	5.0 (3.5)	7.0 (5.4)	4.0 (8.0)	16.0 (4.9)	0.4486
Respiratory symptoms	9.0 (6.3)	5.0 (3.8)	4.0 (8.0)	18.0 (5.6)	0.4833
Gastrointestinal symptoms	6.0 (4.2)	9.0 (6.9)	5.0 (10.0)	20.0 (6.2)	0.1021
Musculoskeletal symptoms	17.0 (11.9)	19.0 (14.6)	13.0 (26.0)	49.0 (15.2)	0.0554 ^++^
Neurological symptoms	11.0 (7.7)	19.0 (14.6)	12.0 (24.0)	42.0 (13.0)	0.0100 ^+++^
Cardiological symptoms	4.0 (2.8)	12.0 (9.2)	9.0 (18.0)	25.0 (7.7)	0.0022 ^++++^
Kidney symptoms	3.0 (2.1)	2.0 (1.5)	2.0 (4.0)	7.0 (2.2)	0.6349
Other symptoms	30.0 (21.0)	32.0 (24.6)	12.0 (24.0)	74.0 (22.9)	0.7597

^a^ Significant difference in age group between Waves 1 and 2 (*p* = 0.0042) and between Waves 1 and 3 (*p* = 0.0021); ^aa^ Acute respiratory failure; ^b^ Decreased level of consciousness; * Significant difference in dyspnea between Waves 1 and 2 (*p* = 0.0019) and between Waves 1 and 3 (*p* < 0.0001); ** Significant difference in cough between Waves 1 and 3 (*p* = 0.004); *** Significant difference in fever between Waves 1 and 2 (*p* = 0.0320); **** Significant difference in desaturation between Waves 1 and 3 (*p* < 0.0005) and between Waves 2 and 3 (*p* < 0.0010); ^#^ Significant difference in diarrhea between Waves 1 and 2 (*p* = 0.0033); ^##^ Significant difference in myalgia between Waves 1 and 2 (*p* = 0.0308) and between Waves 1 and 3 (*p* < 0.0010); ^###^ Significant difference in anosmia between Waves 1 and 3 (*p* = 0.0185) and between Waves 2 and 3 (*p* = 0.0095); ^+^ Significant difference in ageusia between Waves 1 and 2 (*p* = 0.0167) and between Waves 2 and 3 (*p* = 0.0140); ^++^ Significant difference in musculoskeletal symptoms between Waves 1 and 3 (*p* = 0.0232); ^+++^ Significant difference in neurological symptoms between Waves 1 and 3 (*p* < 0.0050); ^++++^ Significant difference in cardiological symptoms between Waves 1 and 2 (*p* = 0.0215) and between Waves 1 and 3 (*p* < 0.0050).

**Table 2:** Clinical information on the comorbidities of patients admitted with COVID-19 at the public hospital in Belém, Pará, Brazil, from February 2020 to April 2022.

	G1 *n* (%)	G2 *n* (%)	G3 *n* (%)	Total *n* (%)	*p*-Value
Comorbidities					
SAH ^a^	75.0 (52.4)	62.0 (47.7)	29.0 (58.0)	166.0 (51.4)	0.4382
DM ^b^	44.0 (30.8)	50.0 (38.5)	17.0 (34.0)	111.0 (34.4)	0.4086
CHF ^c^	6.0 (4.2)	11.0 (8.5)	2.0 (4.0)	19.0 (5.9)	0.2786
Asthma	7.0 (4.9)	40.0 (3.1)	0.0 (0.0)	11.0 (3.4)	0.1130
COPD ^d^	6.0 (4.2)	60.0 (4.6)	3.0 (6.0)	15.0 (4.6)	0.8790
Alzheimer’s disease	7.0 (4.9)	1.0 (0.8)	0.0 (0.0)	8.0 (2.5)	0.0253 *
Sequel brain stroke	5.0 (3.5)	11.0 (8.5)	7.0 (14.0)	23.0 (7.1)	0.0363 **
Drug allergy	4.0 (2.8)	12.0 (9.2)	9.0 (18.0)	25.0 (7.7)	0.0022 ***
Cardiopathies	10.0 (7.0)	9.0 (6.9)	4.0 (8.0)	23.0 (7.1)	0.9667
Pneumopathies	1.0 (0.7)	12.0 (9.2)	0.0 (0.0)	13.0 (4.0)	0.0002 ^+^
Neuropathies	4.0 (2.8)	14.0 (10.8)	5.0 (10.0)	23.0 (7.1)	0.0181 ^++^
Liver diseases	4.0 (2.8)	1.0 (0.8)	2.0 (4.0)	7.0 (2.2)	0.2908
Other comorbidities	30.0 (21.0)	31.0 (23.8)	18.0 (36.0)	79.0 (24.5)	0.1019

^a^ Systemic arterial hypertension; ^b^ Diabetes mellitus; ^c^ Congestive heart failure; ^d^ Chronic obstructive pulmonary disease; * Significant difference in Alzheimer’s between Waves 1 and 2 (*p* = 0.0315) and between Waves 1 and 3 (*p* = 0.0384); ** Significant difference in stroke sequelae between Waves 1 and 3 (*p* = 0.0139); *** Significant difference in drug allergy between Waves 1 and 2 (*p* = 0.0236) and between Waves 1 and 3 (*p* = 0.0009); ^+^ Significant difference in lung diseases between Waves 1 and 2 (*p* < 0.0005) and 2 and 3 (*p* = 0.0043); ^++^ Significant difference in neuropathies between Waves 1 and 2 (*p* = 0.0067).

**Table 3:** Clinical information on the vaccination status of patients admitted with COVID-19 at the public hospital in Belém, Pará, Brazil, from February 2020 to April 2022.

	G1 *n* (%)	G2 *n* (%)	G3 *n* (%)	Total *n* (%)	*p*-Value
Vaccine doses					
0 vaccine dose	143.0 (100.0)	110.0 (84.6)	22.0 (44.0)	275 (85.1)	<0.0001 *
1 vaccine dose	0.0 (0.0)	15.0 (11.5)	3.0 (6.0)	18 (5.6)	
2 vaccine doses	0.0 (0.0)	5.0 (3.8)	18.0 (36.0)	23 (7.2)	
1 booster	0.0 (0.0)	0.0 (0.0)	5.0 (10.0)	5 (1.5)	
2 booster	0.0 (0.0)	0.0 (0.0)	2.0 (4.0)	2 (0.6)	
Total vaccinated	0.0 (0.0)	20.0 (15.4)	28.0 (56.0)	48 (14.9)	

* Significant difference in vaccine doses between Waves 1 and 2 (*p* < 0.0001), Waves 1 and 3 (*p* < 0.0001), and Waves 2 and 3 (*p* < 0.0001).

**Table 4:** Clinical information on ventilatory support and oxygen therapy for patients hospitalized with COVID-19 at the public hospital in Belém, Pará, Brazil, from February 2020 to April 2022.

	G1 *n* (%)	G2 *n* (%)	G3 *n* (%)	Total *n* (%)	*p*-Value
Oxygen therapy					
NC ^a^	100.0 (69.9)	46.0 (35.4)	18.0 (36.0)	164.0 (50.8)	<0.0001 *
NRM ^b^	4.0 (2.8)	45.0 (34.6)	13.0 (26.0)	62.0 (19.2)	<0.0001 **
CN ^c^	3.0 (2.1)	6.0 (4.6)	0.0 (0.0)	9.0 (2.8)	0.0856
VM ^d^	2.0 (1.4)	0.0 (0.0)	0.0 (0.0)	2.0 (0.6)	0.0868
Ventilatory support					
NIV ^e^	0.0 (0.0)	15.0 (11.5)	1.0 (2.0)	16.0 (4.9)	<0.0001 ***
IMV ^f^	41.0 (28.7)	58.0 (44.6)	18.0 (36.0)	117.0 (36.2)	0.0987

^a^ Nasal cannula; ^b^ Non-rebreather mask; ^c^ Continuous nebulization; ^d^ Non-invasive ventilation; ^e^ Non-invasive ventilation; ^f^ Invasive mechanic ventilation; * Significant difference in NC between Waves 1 and 2 (*p* < 0.0001) and between Waves 1 and 3 (*p* 0.0002); ** Significant difference in NRM between Waves 1 and 2 (*p* < 0.0001) and between Waves 1 and 3 (*p* < 0.0001); *** Significant difference in NIV between Waves 1 and 3 (*p* < 0.0001) and between Waves 2 and 3 (*p* = 0.0678).

**Table 5:** Information on the origin and hospital outcome of patients admitted with COVID-19 at the public hospital in Belém, Pará, Brazil, from February 2020 to April 2022.

Hospital Outcome	G1 *n* (%)	G2 *n* (%)	G3 *n* (%)	Total *n* (%)	*p*-Value
Transfer	83.0 (58.0)	87.0 (66.9)	28.0 (56.0)	198.0 (61.3)	0.4400 *
Discharge	25.00 (17.5)	16.0 (12.3)	7.0 (14.0)	48.0 (14.9)	
Death	35.00 (24.5)	27.0 (20.8)	15.0 (30.0)	77.0 (23.8)	

* Significant difference in provenance between Waves 1 and 2 (*p* < 0.0001) and between Waves 2 and 3 (*p* = 0.0002).

## Data Availability

All data referred to in this study are available in the manuscript.
